# The incidence and outcome of hypertension at diagnosis in children with a kidney tumor

**DOI:** 10.1007/s00467-024-06304-w

**Published:** 2024-01-27

**Authors:** Gerrit van den Berg, Paulien A. M. A. Raymakers-Janssen, Roelie M. Wösten-van Asperen, Marry M. van den Heuvel-Eibrink

**Affiliations:** 1 Princess Máxima Center for Pediatric Oncology, Utrecht, The Netherlands; 2 Department of Pediatric Nephrology, Wilhelmina Children’s Hospital/University Medical Center Utrecht, Utrecht, the Netherlands; 3 Department of Pediatric Intensive Care, Wilhelmina Children’s Hospital/University Medical Center Utrecht, Utrecht, the Netherlands; 4 Theme Child Health, Wilhelmina Children’s Hospital/University Medical Center Utrecht, Utrecht, the Netherlands

With great interest we have read the review about hypertension in Wilms tumor [1]. The review notes a substantial range of 22–79% in the prevalence of hypertension at the time of diagnosis. Notably, this hypertension typically was asymptomatic, notwithstanding the existence of severe complications. Unfortunately, our paper on characteristics and outcome of children with Wilms tumor requiring intensive care admission, was not included. In this Dutch cohort study in 5 centers, 7/175 patients were admitted because of hypertension [2]. But, even with this study, characteristics of hypertension at diagnosis are unclear as large cohort studies are lacking.

This research letter shows the incidence, life-threatening symptoms, and clinical outcomes observed in a substantial national retrospective cohort encompassing 147 children with a kidney tumor, in the Princess Máxima Center for Pediatric Oncology, The Netherlands diagnosed between January 1, 2015, and January 1, 2021. Children were considered hypertensive if they had at least three consecutive oscillometric measurements of systolic and/or diastolic blood pressure (BP) > 95th percentile or if they started with antihypertensive medication. Children with BP between 95th percentile and 95th percentile + 12 mmHg were considered to have “grade 1 hypertension” and children with BP ≥ 95th percentile + 12 mmHg were considered to have “grade 2 hypertension” [3].

In this national cohort, 80/147 (55%) of children with a kidney tumor had hypertension at diagnosis, of which most children had hypertension grade 1 (70%) (Table 1). Children with hypertension were younger (31 months, SD 34), compared to children without hypertension (40 months, SD 44) (*p* < 0.05 Mann–Whitney U-test). There were no differences in occurrence of hypertension between girls and boys, nor tumor subtypes. Three patients were admitted to the intensive care due to hypertension. One of these patients had no life-threatening symptoms. The second patient had encephalopathy and seizures, without abnormalities on brain MRI-scan, and also a decreased diastolic heart function without left ventricle hypertrophy. The third patient had left ventricle hypertrophy with normal cardiac function. After treatment with intravenous labetalol and an ACE-inhibitor, all complications of these patients fully recovered.

**Table 1:** Demographics of patients

Variable	All *N* = 147 (%)	HT at diagnosis *N* = 80 (55%)	HT grade I *N* = 48/69* (70%)	HT grade 2 *N* = 21/69 (30%)
Female sex – yes	79 (54)	43 (54)	24 (50)	12 (57)
Age (months), median (IQR)	35 (18–55)	31 (15–48)	33 (18–52)	17 (8–32)
Tumor diagnosis				
- Wilms tumor st I–III	84	49	30	17
- Wilms tumor st IV (including IV + V)	28	14	10	2
- Wilms tumor st V	14	9	4	2
- CMN	5	3	2	
- CN	5	2	1	
- MRTK	2	0		
- RCC	6	3	1	
- Nephrogenic rest	2	0		
- Metanephric adenoma	1	0		
Complications of hypertension				
- PICU admission due to hypertension	3	3		3
- Neurologic symptoms (physical examination, *n* = 147)	1	1		1
- Retinopathy by fundoscopy (*n* = 15)	0			
- Cardiac dysfunction (physical examination (*n* = 147) and echocardiography (*n* = 36))	2	2		2
- Signs of thrombotic microangiopathy (hemolytic anemia and thrombocytopenia, *n* = 147)	0			
- Proteinuria (protein/creatinine ratio > 20 mg/mmol or < 50 mg/mmol in children < 24 months, *n* = 29)	17	17	8	9
Oral antihypertensive medication				
- ACE-inhibitors		34		
- amlodipine		17		
- betablocker		2		
- combination of enalapril and amlodipine		12		
Hypertension 1 month after nephrectomy	31 (21%)	29 (36%)	9 (19%)	16 (76%)
Hypertension at end of tumor treatment	28 (19%)	18 (23%)	3 (6%)	9 (43%)
eGFR at end of tumor treatment, mean (SD)	121 (29)	130 (30)	133 (32)	130 (18)

*HT* hypertension, *IQR* interquartile range, *CMN* congenital mesoblastic nephroma, *CN* cystic nephroma, *MRTK* malignant rhabdoid tumors of the kidney, *RCC* renal cell carcinoma, *PICU* pediatric intensive care unit, *eGFR* estimated glomerular filtration rate by revised Schwartz-formula

*in 11 patients no ‘grade’ was determined (e.g. normal blood pressure after immediate start of antihypertensive medication)

Patients were able to discontinue antihypertensive medication directly after nephrectomy at a rate of 64% (51/80) and, respectively, 77% (62/80) by the end of tumor therapy.

Patients with hypertension at diagnosis had a higher eGFR at end of treatment (mean 130, SD 31), compared to children without hypertension at diagnosis (mean 111, SD 25) (linear regression analysis, *p* < 0.001, even after correction for age and use of hypertensive medication at end of treatment). An increased eGFR may indicate hyperfiltration [4]. To support this hypothesis, it may help to evaluate contralateral kidney volume and have longer-term follow up, measuring blood pressure, proteinuria, and kidney function.

In conclusion, hypertension at diagnosis is frequently observed in children with a kidney tumor. Although 30% of these patients experienced a grade 2 hypertension, the ultimate incidence of life-threatening complications is very low. Hypertension at diagnosis is associated with a higher eGFR at end of treatment, possibly suggesting hyperfiltration, which supports the proposed recommendations for follow-up in the review [1].

## Data Availability

All data generated or analysed during this study are included in this published article.

## References

[1] Hsiao W, Denburg M, Laskin B (2024). Hypertension in Wilms tumor. Pediatr Nephrol.

[2] Steur A, Raymakers-Janssen PAMA, Kneyber MCJ, Dijkstra S, van Woensel JBM, van Waardenburg DA, van de Ven CP, van der Steeg AFW, Wijnen M, Lilien MR, de Krijger RR, van Tinteren H, Littooij AS, Janssens GO, Peek AML, Tytgat GAM, Mavinkurve-Groothuis AM, van Grotel M, van den Heuvel-Eibrink MM, Asperen RMW (2022). Characteristics and outcome of children with wilms tumor requiring intensive care admission in first line therapy. Cancers (Basel).

[3] Flynn JT, Kaelber DC, Baker-Smith CM, Blowey D, Carroll AE, Daniels SR, de Ferranti SD, Dionne JM, Falkner B, Flinn SK, Gidding SS, Goodwin C, Leu MG, Powers ME, Rea C, Samuels J, Simasek M, Thaker VV, Urbina EM; Subcommittee on Screening and Management of High Blood Pressure in Children (2017) Clinical practice guideline for screening and management of high blood pressure in children and adolescents. Pediatrics 140:e20171904. 10.1542/peds.2017-1904 10.1542/peds.2017-1904 28827377

[4] Cortinovis M, Perico N, Ruggenenti P, Remuzzi A, Remuzzi G (2022). Glomerular hyperfiltration. Nat Rev Nephrol.

